# Human Granulocytic Anaplasmosis Acquired in Scotland, 2013

**DOI:** 10.3201/eid2006.131849

**Published:** 2014-06

**Authors:** Peter Hagedorn, Maren Imhoff, Christian Fischer, Cristina Domingo, Matthias Niedrig

**Affiliations:** Robert Koch-Institut, Berlin, Germany (P. Hagedorn, M. Imhoff, C. Domingo, M. Niedrig);; Charité-Universitätsmedizin Berlin, Berlin (C. Fischer)

**Keywords:** Human granulocytic anaplasmosis, Anaplasma phagocytophilum, Scotland, vector-borne infections, bacteria

**To the Editor:** Human granulocytic anaplasmosis is a tick-borne disease caused by *Anaplasma phagocytophilum,* an obligate intracellular gram-negative bacterium that infects granulocytes. The usual clinical signs and symptoms include nonspecific fever, chills, headache, and myalgia. Infection is usually mild or asymptomatic, but severe systemic complications can occur, leading to a need for intensive care and estimated fatality rates of 0.5%–1.0% (*1*,*2*).

*A. phagocytophilum* was first described in 1932 in Scotland as the causative agent of tick-borne fever in sheep (*3*). Although some clinical cases of human granulocytic anaplasmosis have been reported in Europe, mostly from Slovenia, Sweden, and Poland (*4*), most cases have occurred in the United States. This difference cannot be explained by the prevalence of the pathogen in ticks or human exposure to the pathogen because the 3% prevalence of *A. phagocytophilum* among *Ixodes ricinus* ticks in Europe seems to be nearly as high as that among ticks in the United States (*2*). The median seroprevalence rate for *A. phagocytophilum* infection among humans in Europe is 6.2%, reaching up to 21% (*2*). This incongruence between seroprevalence rate and number of human cases might be associated with underdiagnosis of cases (*2*), a high rate of asymptomatic disease (*5*), or cross-reactivities in serologic tests that might lead to overestimation of seroprevalence rate (*5*).

In August 2013, an immunocompetent 40-year-old man sought treatment for fever (≈39°C) and other nonspecific symptoms such as malaise, myalgia, and severe headache 3 days after becoming aware of several tick bites received while on a hiking vacation in Scotland. The man had removed the 3 tick nymphs from his legs immediately after their discovery and stored them in a plastic container; they were later sent to the Consultant Laboratory for Tick-borne Encephalitis in Berlin, Germany, for analysis. When the patient returned to Germany, 5 days after the onset of symptoms and 8 days after tick removal, a blood sample was collected (sample 1) and the bite sites were swabbed with a sterile cotton bud. By that time, the fever was gone, but malaise and other symptoms persisted. The patient began taking doxycycline, and within 2 days all symptoms subsided and the patient recovered completely.

A second blood sample was collected 28 days after tick removal (sample 2). Complete blood counts and chemistry panels were performed for both samples. All values were within the reference range except that for lactate dehydrogenase (248 U/L), which was moderately increased over the baseline value of <245 U/L in sample 1. Values did not differ substantially between the 2 samples.

DNA from whole-blood samples and swabs was extracted (QIAamp DNA Blood Mini Kit; QIAGEN, Hilden, Germany) and tested for *A. phagocytophilum*, *Babesia* spp., *Borrelia* spp., and *Rickettsia* spp. by using commercially available rapidSTRIPE assays for *Anaplasma*, *Babesia*, *Borrelia*, *Rickettsia* (all Analytik Jena AG; Jena, Germany). DNA extracted from blood and swab samples was negative for all tested pathogens.

After the tick specimens were taxonomically identified as *I. ricinus*, DNA/RNA was extracted (blackPREP Tick DNA/RNA Kit; Analytik Jena AG) and tested for the same pathogens. All 3 ticks were negative for *Babesia* spp., *Borrelia* spp., and *Rickettsia* spp., but 2 were positive for *A. phagocytophilum.*

Indirect immunofluorescence assays (Focus Diagnostics, Cypress, CA, USA) performed on the paired serum samples revealed an increased *A. phagocytophilum*–specific IgM titer, from 20 at 5 days after symptom onset to 80 at 20 days later; the *A. phagocytophilum*–specific IgG titer rose from a high titer of 800 to >3,200 over this period.

The presence and 4-fold increase of *A. phagocytophilum*–specific IgM and IgG in paired serum samples confirmed the diagnosis of human granulocytic anaplasmosis in accordance with Centers for Disease Control and Prevention criteria (*6*). As described previously for several cases of human granulocytic anaplasmosis, patient blood counts were within reference limits but serum lactate dehydrogenase level was elevated (*7*). The diagnosis was further corroborated by detection of *A. phagocytophilum* DNA in 2 of the 3 ticks removed from the patient’s skin. PCR amplification failed to detect *A. phagocytophilum* DNA in the patient’s blood, consistent with previous studies documenting frequent lack of *A. phagocytophilum* DNA detection in whole blood and a substantial drop in PCR positivity after the acute phase of illness (*8*).

Human granulocytic anaplasmosis is not usually reported in Scotland like it is in the rest of Europe. The case originated from an area with long-established disease occurrence in ruminants, but the literature reports only 1 case of human infection in southwestern Scotland (*9*), ≈500 km from where this infection was probably acquired. Correct diagnosis would have been difficult had the patient not conserved the ticks and contacted the Consultant Laboratory for Tick-borne Encephalitis immediately after returning to Germany. A large number of human granulocytic anaplasmosis cases might be missed because general practitioners may not be aware of the pathogen’s existence or its distribution.

## References

[1] Bakken JS, Dumler JS. Clinical diagnosis and treatment of human granulocytotropic anaplasmosis. Ann N Y Acad Sci. 2006;1078:236–47 . 10.1196/annals.1374.042 17114714

[2] Dumler JS, Choi KS, Garcia-Garcia JC, Barat NS, Scorpio DG, Garyu JW, Human granulocytic anaplasmosis and *Anaplasma phagocytophilum.* Emerg Infect Dis. 2005;11:1828–34 . 10.3201/eid1112.050898 16485466 PMC3367650

[3] Woldehiwet Z. The natural history of *Anaplasma phagocytophilum.* Vet Parasitol. 2010;167:108–22. 10.1016/j.vetpar.2009.09.013 19811878

[4] Blanco JR, Oteo JA. Human granulocytic ehrlichiosis in Europe. Clin Microbiol Infect. 2002;8:763–72. 10.1046/j.1469-0691.2002.00557.x 12519349

[5] Graf PC, Chretien JP, Ung L, Gaydos JC, Richards AL. Prevalence of seropositivity to spotted fever group rickettsiae and *Anaplasma phagocytophilum* in a large, demographically diverse US sample. Clin Infect Dis. 2008;46:70–7. 10.1086/524018 18171216

[6] Centers for Disease Control and Prevention. Case definitions for infectious conditions under public health surveillance. MMWR Recomm Rep. 1997;46(RR-10):1–55 . 9148133

[7] Walder G, Fuchs D, Sarcletti M, Berek K, Falkensammer B, Huber K, Human granulocytic anaplasmosis in Austria: epidemiological, clinical, and laboratory findings in five consecutive patients from Tyrol, Austria. Int J Med Microbiol. 2006;296(Suppl 40):297–301. 10.1016/j.ijmm.2005.12.001 16531117

[8] Bakken JS, Aguero-Rosenfeld ME, Tilden RL, Wormser GP, Horowitz HW, Raffalli JT, Serial measurements of hematologic counts during the active phase of human granulocytic ehrlichiosis. Clin Infect Dis. 2001;32:862–70. 10.1086/319350 11247709

[9] Sumption KJ, Wright DJ, Cutler SJ, Dale BA. Human ehrlichiosis in the UK. Lancet. 1995;346:1487–8. 10.1016/S0140-6736(95)92502-3 7491006

